# Cell‐free synthesis of isotopically labelled peptide ligands for the functional characterization of G protein‐coupled receptors

**DOI:** 10.1002/2211-5463.12008

**Published:** 2015-12-29

**Authors:** Lisa Joedicke, Raphael Trenker, Julian D. Langer, Hartmut Michel, Julia Preu

**Affiliations:** ^1^ Department of Molecular Membrane BiologyMax Planck Institute of BiophysicsFrankfurt am MainGermany; ^2^ Structural Biology DivisionThe Walter and Eliza Hall Institute of Medical Research1G Royal ParadeParkvilleVIC3052Australia; ^3^ Department of Medical BiologyThe University of MelbourneParkvilleVIC3052Australia

**Keywords:** cell‐free protein production, disulphide bonds, G protein‐coupled receptor, MALDI‐TOF, Radioligand‐binding assay

## Abstract

Cell‐free systems exploit the transcription and translation machinery of cells from different origins to produce proteins in a defined chemical environment. Due to its open nature, cell‐free protein production is a versatile tool to introduce specific labels such as heavy isotopes, non‐natural amino acids and tags into the protein while avoiding cell toxicity. In particular, radiolabelled peptides and proteins are valuable tools for the functional characterization of protein–protein interactions and for studying binding kinetics. In this study we evaluated cell‐free protein production for the generation of radiolabelled ligands for G protein‐coupled receptors (GPCRs). These receptors are seven‐transmembrane‐domain receptors activated by a plethora of extracellular stimuli including peptide ligands. Many GPCR peptide ligands contain disulphide bonds and are thus inherently difficult to produce in bacterial expression hosts or in *Escherichia coli*‐based cell‐free systems. Here, we established an adapted *E. coli*‐based cell‐free translation system for the production of disulphide bond‐containing GPCR peptide ligands and specifically introduce tritium labels for detection. The bacterial oxidoreductase DsbA is used as a chaperone to favour the formation of disulphide bonds and to enhance the yield of correctly folded proteins and peptides. We demonstrate the correct folding and formation of disulphide bonds and show high‐affinity ligand binding of the produced radio peptide ligands to the respective receptors. Thus, our system allows the fast, cost‐effective and reliable synthesis of custom GPCR peptide ligands for functional and structural studies.

**Abbreviations**
[^3^H]‐C5a^CF^: cell‐free‐synthesized tritium‐labelled complement 5a
[^3^H]‐pET‐1^CF^: furin‐processed cell‐free‐synthesized tritium‐labelled endothelin‐1
C5a^CF^: cell‐free‐synthesized complement 5a
fET‐1^CF^: full‐length cell‐free‐synthesized endothelin‐1
FM: feeding mix
GPCR: G protein‐coupled receptor
GSH: reduced glutathione
IMAC: immobilized metal ion affinity chromatography
IPTG: Isopropyl β‐D‐thiogalactopyranoside
pET‐1^CF^: furin‐processed cell‐free‐synthesized endothelin‐1
RM: reaction mix

Cell‐free protein production is a versatile tool for the recombinant production of proteins in a predetermined chemical surrounding. The major advantage of cell‐free protein synthesis is the possibility to control nearly all steps of protein production. The open nature of cell‐free systems allows the incorporation of tags, non‐natural or labelled amino acids while avoiding the problem of toxic cellular events during recombinant protein production 1. In general, cell free production systems are suitable to generate milligram quantities of proteins per millilitre of reaction mixture. These yields can be achieved by optimizing all steps of protein production such as the incorporation of T7 terminators into the DNA sequence, or the preparation of extracts from RNase deficient *Escherichia coli* strains and bacteria with engineered pathways of the central metabolism to enhance protein production 2, 3. Mostly, cell‐free reactions are performed in batch, fed‐batch or continuous exchange mode 4, whereas coupled systems combining translation and transcription yield highest protein amounts, but require the addition of RNA polymerases encoded by bacteriophages 5. Since the first description of cell‐free protein synthesis by Nirenberg and Matthaei 1 various applications have evolved, including the synthesis of therapeutics and pharmaceutical proteins, membrane proteins and virus‐like particles 2, 5, 6, 7, 8, 9. By now, cell‐free protein synthesis has been optimized to allow manufacturing scale protein production 2 as well as high throughput production of protein libraries in the nanoliter scale 10.

Cell‐free systems were first described using *E. coli* as source to extract all necessary components for protein production 1. *E. coli* extracts are fast and easily produced, whereas for target proteins requiring complex folding and post‐translational modifications, eukaryotic sources such as rabbit reticulocytes, yeasts, insect cells and wheat germ 4, 11, 12, 13 are widely used. Post‐translational modifications may also be introduced using the recently developed PURE (protein expression using recombinant elements) system, which does not depend on cellular extracts but relies on a recombinantly produced and purified protein translation machinery 14, 15. Especially when using bacterial extracts for cell‐free synthesis, the formation of disulphide bonds to ensure correct folding of the target protein can be challenging. The problem may be overcome by the addition of chaperones, disulphide bond isomerases or oxidoreductases. In particular, the bacterial Dsb system consisting of several disulphide isomerases and oxidoreductases has been employed to favour disulphide bond formation when using bacterial systems 2, 8, 16.

Here, we describe a modified *E. coli*‐based continuous exchange cell‐free system to synthesize small proteins and peptides containing disulphide bonds and demonstrate their application in radioligand‐binding assays to functionally characterize G protein‐coupled receptors (GPCRs). GPCRs represent the largest family of cell surface receptors in higher eukaryotes. They are key regulators of cellular communication and respond to a variety of extracellular stimuli including protein and peptide ligands. 17 GPCR peptide ligands mostly derive from high molecular weight protein precursors and are secreted into the bloodstream 18. To maintain their structural integrity and to ensure correct proteolytic processing from their precursors, these ligands often contain disulphide bonds 19. Complement 5a (C5a) and endothelin‐1 (ET‐1) are two high‐affinity ligands activating G protein‐coupled receptors. The C5a anaphylatoxin plays crucial roles in the activation of the innate immune response and the initiation of sepsis by binding to the complement 5a receptor (C5aR). C5a is generated from complement 5 by proteolytic processing and is secreted into the bloodstream. Here, it exerts its function by activating signalling pathways through the C5aR which is mainly expressed in cells of myeloid origin. 20 C5a is a 74 amino acid protein, composed of four α‐helices that are connected by three intramolecular cysteine bridges 21 which are indispensable for high‐affinity binding to the C5aR 22. In addition to the six bridged cysteine residues the protein contains one reduced cysteine 21.

Endothelin‐1 is a 21 amino acid peptide activating the endothelin A and B receptors (ET_A_R and ET_B_R respectively). ET‐1 is a potent vasoconstrictor 23 but also functions in many nonvascular tissues and processes 24. The peptide contains two disulphide bonds, which are necessary for its correct proteolytical processing from big ET‐1 19 as well as its high‐affinity binding to the ET_A_R and ET_B_R 25.

The interactions of C5a with the C5aR and functional aspects of ET‐1 binding to the ET_A_R and ET_B_R have been intensively characterized using ^125^I‐labelled versions of the peptide ligands. So far, long half‐life tritium labelled variants of the peptides are unavailable as chemically introduced tritium labels require reducing conditions which are incompatible with cysteine bond formation. Here, we report the fast, reliable and cost‐effective cell‐free synthesis of tritium‐labelled C5a and endothelin‐1 using an adapted *E. coli*‐based cell‐free protein production system. We demonstrate the correct formation of the cysteine bonds in each ligand and show high‐affinity binding of both peptides towards their endogenous receptors.

## Results

In this study we aimed at producing tritium labelled peptide ligands for the functional characterization of GPCRs. We chose complement 5a and endothelin‐1 as model ligands as they display different folds, numbers of disulphide bonds and binding modes. We show the correct folding and formation of disulphide bonds for both ligands and demonstrate high‐affinity binding to their respective endogenous receptors.

### Cell‐free translation of disulphide‐bonded peptide ligands

**Figure 1:**
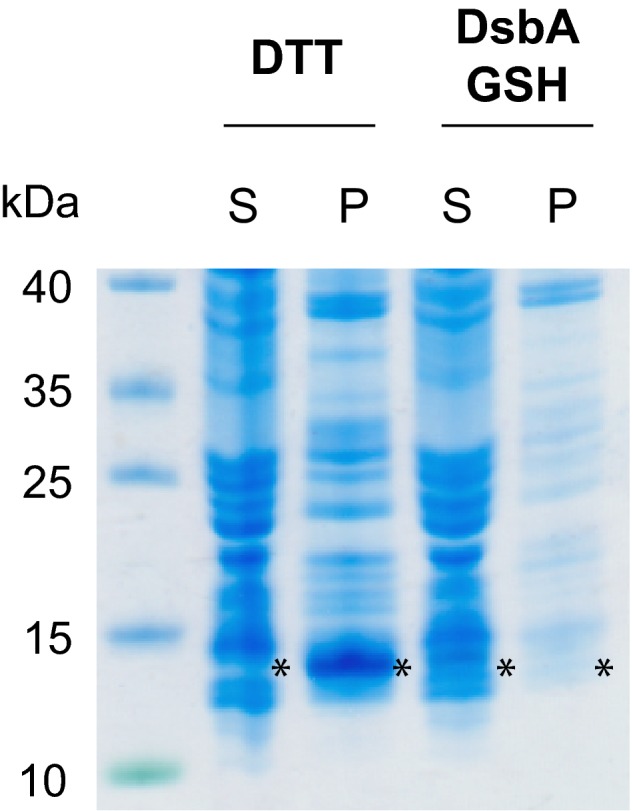
Complement 5a can be produced in soluble form in the presence of DsbA and GSH indicating correct folding and disulphide bond formation. Under reducing conditions (DTT) cell‐free produced C5a is insoluble and can only be detected in the pellet fraction of the reaction mix (P, indicated with an asterisk). Upon addition of DsbA and GSH the amount of peptide produced is decreased, but C5a^CF^ can be detected in the soluble fraction (S, asterisk) indicating correct folding and cysteine bond formation. Abbreviations: S, soluble fraction; P, precipitate fraction.

Complement 5a and endothelin‐1 are two high‐affinity GPCR ligands that depend on the correct formation of cysteine bonds to bind and fully activate their respective receptors. Generating disulphide bonds in newly synthesized proteins in cell‐free systems is challenging as cell‐free protein translation generally requires reducing conditions to maintain the integrity of the translation machinery 16. A reductive environment in cell‐free systems is usually ensured by the addition of dithiothreitol (DTT) to the reaction mixture. Cell‐free translations of C5a and ET‐1 in the presence of DTT resulted in strong precipitation (Fig. 1), indicating the production of misfolded proteins. Therefore, we used the bacterial disulphide oxidoreductase DsbA as a chaperone to favour the generation of disulphide bonds and replaced DTT with reduced glutathione (GSH) which possesses a lower redox potential than DTT 26, 27. StrepII‐tagged DsbA was produced in *E. coli* and purified using StrepTactin. The protein purity was analysed by SDS/PAGE and no contaminating proteins could be detected (Fig. S1). When DsbA and GSH were present, cell‐free translations of C5a and ET‐1 did not display any precipitates, pointing towards a successful formation of disulphide bonds.

**Figure 2:**
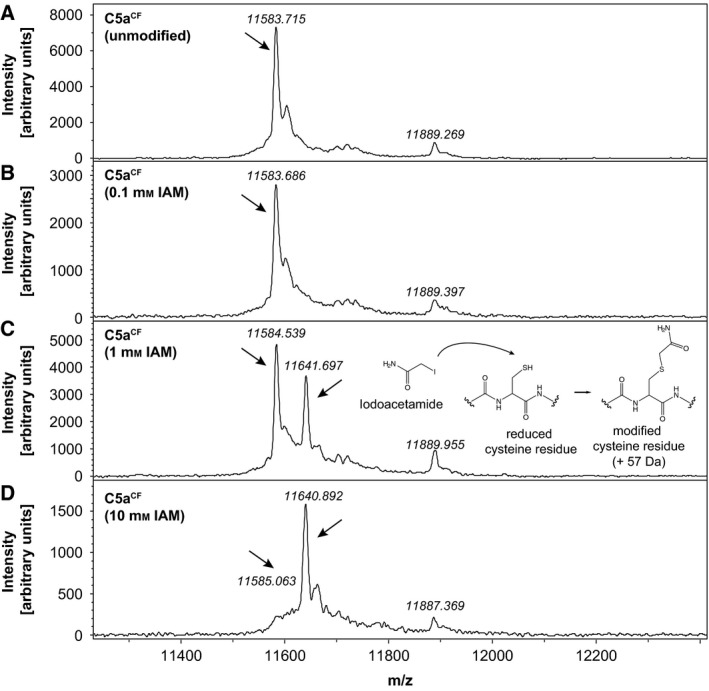
Iodoacetamide (IAM) labelling of C5a^CF^ proofs the existence of only one free cysteine residue. (A) C5a^CF^ containing an N‐terminal 6xHis tag and TEV cleavage site is detected in MALDI‐TOF at 11583 Da corresponding to the theoretical molecular weight of 11582 Da. (B) Labelling with 0.1 mm IAM does not result in any mass shift of the protein. (C and D) Labelling with 1 mm and 10 mm IAM sequentially shifts the molecular weight of C5a^CF^ towards 11640 Da corresponding to a 57 Da mass shift expected for one accessible sulphydryl group. Nonspecific side reactions are not observed as no additional peaks at higher molecular weight are detected with increasing concentrations of IAM.

To assess whether disulphide bonds were formed correctly in our adapted cell‐free system, we evaluated the number of free sulphydryl groups in C5a^CF^ and ET‐1^CF^. C5a contains seven cysteine residues of which six form disulphide bonds (Cys^21^‐Cys^47^, Cys^22^‐Cys^54^, Cys^34^‐Cys^55^). Cys_27_ remains unbound and displays a reduced sulphydryl moiety 21, 28. Iodoacetamide (IAM) labelling was used to probe free sulphydryl groups in C5a^CF^. Each incorporated IAM moiety results in the addition of 57 Dalton per free sulphydryl group to the molecular mass. Changes in the molecular mass of C5a^CF^ and ET‐1^CF^ were detected using matrix‐assisted laser desorption ionization‐time of flight mass spectrometry (MALDI‐TOF). Prior to these analyses, the identities of both peptides were verified using fingerprinting of proteolytic digests and liquid chromatography‐coupled tandem mass spectrometry (data not shown). C5a^CF^ (containing the N‐terminal 6xHis tag and TEV cleavage site) is detected at a molecular weight of 11583 Da which is in good agreement with the theoretical molecular weight of 11582 Da (Fig. 2A). Labelling the protein with 0.1, 1 and 10 mm IAM sequentially shifts the molecular weight by 57 to 11640 Da, corresponding to the labelling of one free sulphydryl group (Fig. 2B–D). Nonspecific side reactions with amino acids other than cysteine were not observed as no higher molecular weight derivatives of C5a^CF^ were detected with increasing IAM concentrations. The presence of only one modifiable sulphydryl group indicates the correct formation of three intramolecular cysteine bridges in C5a^CF^.

### Functional characterization of cell‐free produced C5a

**Figure 3:**
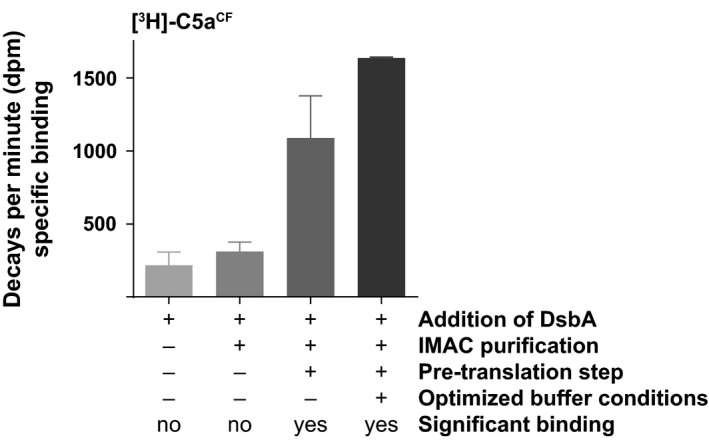
Optimization of the cell‐free translation conditions of [^3^H]‐C5a^CF^ results in significant and specific binding to the C5aR. Specific binding of [^3^H]‐C5a^CF^ was assessed after producing the peptide in the presence of DsbA and after a subsequent IMAC purification step (left bars). Due to high nonspecific background, specific binding is low and nonsignificant. Upon the introduction of the pretranslation step to remove endogenously present proline, specific binding of [^3^H]‐C5a^CF^ is strongly increased (dark grey bar) and statistically significant. Additional optimization of the assay buffer conditions (addition of NaCl to the assay and wash buffer) further increases the detected specific binding of [^3^H]‐C5a^CF^ (black bar).

To assess whether the disulphide bonds in C5a^CF^ were formed in native manner, the protein was translated in tritiated form to test its capability of binding to the complement 5a receptor (C5aR). However, cell‐free translated C5a ([^3^H]‐C5a^CF^) displayed only low and nonsignificant binding to the C5aR in the first translation trials (Fig. 3). When incorporating labelled amino acids into proteins translated with *E. coli*‐based cell‐free systems, isotope dilution due to the presence of endogenous amino acids in the S30 extract can occur 29. In the case of synthesizing a radioligand for functional studies on GPCRs, a dilution of the tritium‐labelled amino acid with an endogenous unlabelled variant results in a mixture of fully or partially labelled and unlabelled ligand. This inhomogeneous mixture of ligand species complicates the calculation of the concentration of the radioligand and thereby compromises the determination of binding constants. We removed endogenously present amino acids in our S30 extract by a pretranslation step. The cell‐free reaction is started with all necessary components present, omitting the amino acid that is to be introduced in labelled version. During this incubation the endogenous amino acid is incorporated into an unlabelled ligand which can then be removed by its affinity tag. Using this strategy we could obtain fully functional [^3^H]‐C5a^CF^ binding specifically to the C5aR and displaying low nonspecific background (Fig. 3). To verify the incorporation rate of labelled proline residues into C5a^CF^, we translated the peptide in the presence of uniformly labelled ^15^N‐^13^C‐proline and determined an incorporation rate of higher than 80% using MALDI‐MS (Fig. S4).

**Figure 4:**
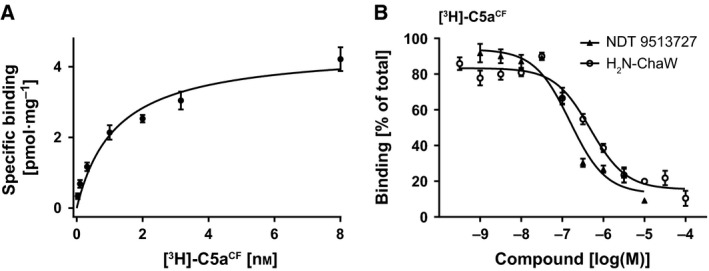
Binding parameters of [^3^H]‐C5a^CF^ towards the complement 5a receptor. (A) [^3^H]‐C5a^CF^ binds with high affinity (K_D_ 1.18 ± 0.27 nm) and in saturable manner to the C5aR. (B) The peptide antagonist H_2_N‐ChaW displaces [^3^H]‐C5a^CF^ with an inhibition constant of 169.1 ± 27.7 nm (open circles). The small molecule inverse agonist NDT 9513727 displays an inhibition constant of 54.5 ± 9.9 nm (triangles).

As the interaction between C5a and its receptor is mainly mediated by the disulphide bridged core region of C5a (residues 21–55, 22) and the C‐terminal region (especially Arg^74^ 30) the N‐terminal hexa‐histidine tag was not removed. The cell‐free synthesized ligand binds to the complement 5a receptor with a dissociation constant of 1.18 ± 0.27 nm (Fig. 4A), which corresponds to values determined by Gerard and Gerard for C5aR in native human tissues (1 nm 31, Table 1). Competition experiments with the unlabelled small molecule inverse agonist NDT 9513727 and the peptide antagonist H_2_N‐ChaW resulted in inhibition constants of 169.1 ± 27.7 and 54.5 ± 9.9 nm, respectively (Fig. 4B), confirming that [^3^H]‐C5a^CF^ can be displaced from C5aR with K_I_ values comparable to the literature (Table 1). Thus, cell‐free translated [^3^H]‐C5a^CF^ binds to the C5aR with binding parameters correlating to values determined in native human tissues and with commercially available ^125^I‐C5a, confirming that our cell‐free translation system yields a fully folded and functional radioligand.

**Table 1:** Binding parameters of cell‐free synthesized radioligands

	K_D_ [nm]	Literature K_D_ [nm]		K_I_ [nm]	Literature IC_50_ [nm]
[^3^H]‐C5a^CF^	1.18 ± 0.27	1 31	H_2_N‐ChaW	169.1 ± 27.7 (NMe‐ChaW)	70 52
			NDT 9513727	54.5 ± 9.9	11.6 ± 1.0 53
[^3^H]‐pET‐1^CF^	0.8 ± 0.3	0.33 36	Endothelin‐1	0.24 ± 0.08	1.6 37

### Cell‐free production of endothelin‐1

To further demonstrate the utility of cell‐free production of GPCR ligands, we chose endothelin‐1, a 21 amino acid peptide (2491 Da) containing two disulphide bridges (Cys^1^–Cys^15^, Cys^3^–Cys^11^) 32. Due to the two disulphide bonds, ET‐1 possesses a conformationally constrained N‐terminal region and a flexible, hydrophobic C‐terminal tail 33. Therefore, the N‐terminal hexa‐histidine tag of fET‐1^CF^ was removed by furin cleavage to avoid steric or electrostatic interference with high‐affinity binding to the ET_B_R. Furin endogenously processes pre–pro‐endothelin to big‐endothelin, which is then further converted through C‐terminal cleavage by endothelin‐converting enzymes to endothelin‐1 34, 35. The efficiency of furin cleavage was monitored using MALDI‐TOF spectrometry. Full‐length fET‐1^CF^ containing the N‐terminal tags has a molecular weight of 6949 Da which is expected to decrease upon furin cleavage to 2491 Da for processed pET‐1^CF^.

**Figure 5:**
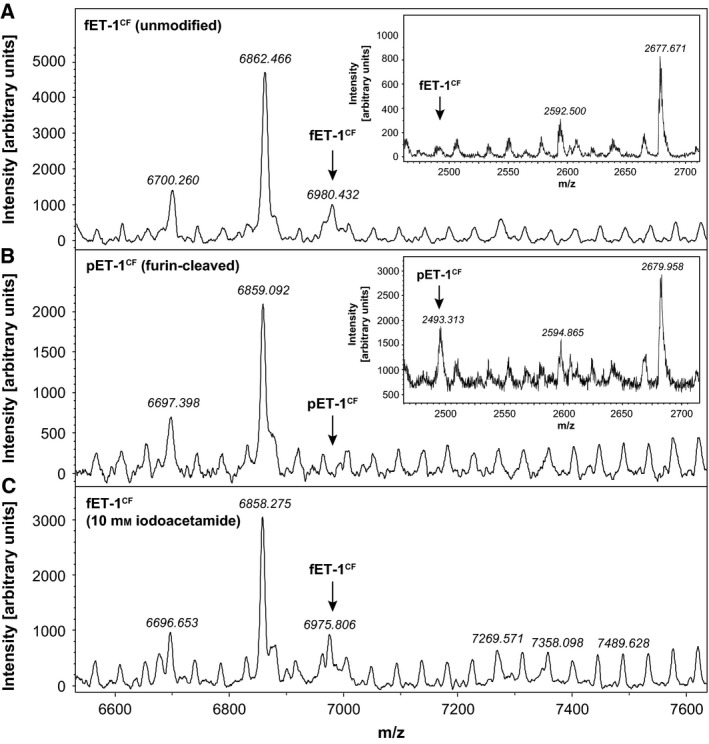
ET‐1^CF^ does not contain free cysteine residues and can be fully processed by furin protease. (A) Full‐length ET‐1^CF^ is detected at 6980 Da and is presumably formylated at the N‐terminal methionine residue (mass addition of 27 Da). (B) Upon treatment with furin, the peptide peak at 6980 Da can no longer be observed, indicating a complete processing of the peptide precursor. Consistently, a signal at 2493 Da corresponding to the cleaved peptide (pET‐1^CF^) can be detected in furin‐treated fET‐1CF but not in uncleaved pET‐1^CF^ (insets in B and A respectively). (C) Treatment of fET‐1^CF^ with 10 mm iodoacetamide does not shift the molecular mass of the peptide, demonstrating that no free cysteine residues are present in the peptide.

Full‐length cell‐free‐synthesized endothelin‐1 is detected as a single peak in the range of 6975 Da in MALDI‐TOF (Fig. 5A). The mass shift of 26 Da compared to the theoretical molecular weight is presumably caused by the formation of two disulphide bridges (−4 Da) and by the formylation of the N‐terminal methionine residue (+27 Da), resulting in a total mass shift of 23 Da. After treatment of fET‐1^CF^ with furin, the peak at 6975 Da can no longer be detected, indicating a full processing of fET‐1^CF^ to pET‐1^CF^ (Fig. 5B). The mass range of the processed peptide (2495 Da) does not display any signals in the unprocessed fET‐1^CF^ samples (Fig. 5A, inset). Upon furin treatment, a signal at 2493 Da can be identified which represents processed pET‐1^CF^ (Fig. 5B, inset). The quality of the MALDI spectra in the range of fET‐1^CF^ is limited by the presence of fragmented PEG8000 molecules which are used in cell‐free reactions to mimic the viscosity of the cytoplasm and cannot be fully removed during the purification process. Although the signal to noise ratio of the spectra is low, these findings indicate the efficient removal of the N‐terminal tags from fET‐1^CF^ and the production of a native‐like pET‐1^CF^ for further functional analysis.

**Figure 6:**
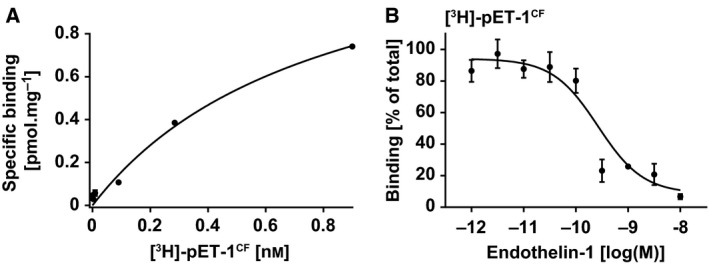
High‐affinity binding of [^3^H]‐pET‐1^CF^ to the endothelin B receptor. (A) [^3^H]‐pET‐1^CF^ binds to the ET_B_R with a dissociation constant of 0.8 ± 0.3 nm. Representative experiment of two experiments performed in duplicate. (B) Unlabelled endothelin‐1 competes with [^3^H]‐pET‐1^CF^ with an inhibition constant of 0.24 ± 0.08 nm.

To assess the formation of cysteine bridges in fET‐1^CF^, free sulphydryl groups were probed with 10 mm iodoacetamide according to the protocol developed for C5a^CF^ (Fig. 2). Iodoacetamide treatment does not result in detectable mass shifts of fET‐1^CF^ indicating that no free sulphydryl groups are present and all cysteine residues form disulphide bridges (Fig. 5C). As these findings indicate the correct folding and processing of ET‐1^CF^, the peptide was produced in tritium‐labelled form to assess its binding parameters to the endothelin B receptor. Due to the small size of [^3^H]‐pET‐1^CF^ (21 amino acids) and the low number of redundant amino acids, we used phenylalanine as labelled amino acid as it displays high‐specific activities and therefore ensures that the peptide can be detected in radioligand‐binding assays. We tested the furin‐cleaved [^3^H]‐pET‐1^CF^ radioligand in binding assays with membranes containing the endothelin B receptor. The cell‐free produced ligand binds to the ET_B_R with high‐affinity resulting in a dissociation constant of 0.8 ± 0.3 nm (Fig. 6A), corresponding to values determined with ^125^I‐ET‐1 (0.33 nm 36) on receptors from native human tissues. Accordingly, homologous competition experiments with unlabeled ET‐1 yielded and inhibition constant of 0.24 ± 0.08 nm (Fig. 6B) which is in good agreement with literature values (1.6 nm 37). We can thus conclude that our cell‐free synthesized radiolabelled endothelin‐1 is a fully functional ligand for the endothelin B receptor which emphasizes the usability of our adapted cell‐free system for the generation of GPCR peptide ligands for functional studies.

## Discussion

Peptide ligands activating G protein‐coupled receptors often contain disulphide bonds which play crucial roles for their stability, tertiary structure and correct biological function. Disulphide bridges also play important roles in the proteolytic processing of peptides from high molecular weight protein precursors 19 and stabilize them upon secretion to avoid proteolytic degradation 18. Therefore, it is not surprising that cysteine residues forming disulphide bonds are among the most conserved amino acids found in proteins 38. We chose complement 5a and endothelin‐1, two high‐affinity GPCR ligands relying on the correct formation of disulphide bonds for their biological function 35, 39 as model peptides to demonstrate a fast and reliable approach for the cell‐free synthesis of custom labelled radioligands for functional studies.

Producing small proteins containing disulphide bonds is inherently difficult in bacterial and cell‐free expression systems, resulting in the engineering of bacterial strains for enhanced disulphide bond formation and the addition of chaperones to cell‐free systems 40, 41. Translation of C5a and ET‐1 in cell‐free systems without adaptions to favour disulphide bond formation resulted in heavy precipitation and low amounts of recombinantly produced protein (Fig. 1). The formation of disulphide bonds in cell‐free systems can be enhanced by the addition of eukaryotic protein disulphide isomerases from the endoplasmatic reticulum and other chaperones such as DnaK, DnaJ and GrpE or GroEL and GroES 42. Furthermore, the bacterial Dsb system has also been employed to promote the formation of disulphide bonds in cell‐free systems. The Dsb system comprises several proteins crucial for disulphide bond formation and isomerization in the bacterial periplasm. Among the Dsb proteins, DsbA is the major disulphide bond donor and displays one of the highest redox potentials 43. However, when DsbA was used to favour the formation of disulphide bonds in antibody fragments translated in *E. coli*‐based cell‐free systems its addition did not improve protein yield and functionality 42. Therefore, the disulphide isomerase DsbC together with other chaperones is often introduced into cell‐free systems to aid the formation of nonconsecutive disulphide bonds 41.

In our system the addition of DsbA combined with the exchange of DTT by reduced glutathione, resulted in an increased yield of soluble and folded C5a^CF^ and ET‐1^CF^. DsbA facilitates the formation of the correct number of disulphide bonds as we demonstrate by iodoacetamide (IAM) labelling and subsequent detection of mass shifts in MALDI‐TOF. Native C5a comprises three cysteine bridges (Cys^21^–Cys^47^, Cys^22^–Cys^54^, Cys^34^–Cys^55^) and one cysteine residue present in reduced form (Cys^27^) 21, 28. C5a^CF^ contains only one IAM reactive moiety as a sequential 57 kDa mass shift is detected with increasing IAM concentrations (Fig. 2). Accordingly, fET‐1^CF^ is unreactive to IAM as it does not contain any unbridged cysteine residues (Fig. 5). Typical nonspecific side reactions such as labelling of terminal amino groups, histidine or lysine side chains 44 can be excluded, as no multiple peaks are detected at higher molecular weights for both proteins. Additionally, controls with lysozyme that contains four disulphide bonds and no free cysteine residue 45, 46 did not yield any nonspecifically labelled species (Fig. S3). We can therefore conclude that the detected mass shift in C5a^CF^ results from one single cysteine residue and that ET‐1^CF^ contains no reduced cysteines, indicating the formation of the correct number of disulphide bridges in both proteins.

The open nature of cell‐free systems facilitates the introduction of non‐natural or labelled amino acids into the sequence of the target protein. Introducing heavy isotope labelled amino acids has been widely employed for NMR spectroscopy studies yielding high amounts of labelled protein 47, 48. When labelling proteins for NMR studies or with tritium‐labelled amino acids for functional studies, a high incorporation efficiency of the labelled amino acids has to be achieved. Full incorporation efficiency can be compromised by the presence of endogenous amino acids in the S30 extract which result in a dilution of the isotope‐labelled amino acid. The S30 extract can further be purified by gel filtration, removing any endogenous amino acids and low molecular weight components. 29 When producing tritium‐labelled peptide ligands for functional studies on GPCRs, it is crucial to obtain an incorporation rate close to 100%, otherwise the functional assays are biased by the presence of unlabelled ligands. Furthermore, contaminations with unlabelled ligand impede the calculation of the concentration of the translated radioligand. We estimated the presence of endogenous amino acids in our extract by translating C5a^CF^ in the absence of proline and detected a substantial amount of protein produced (Fig. S2). Removal of the endogenous amino acids by incubating the cell‐free reaction in the absence of proline and then extracting the produced C5a^CF^ by IMAC resulted in a fully functional radioligand. We analysed the incorporation rate of labelled proline residues and thus the efficiency of the pretranslation step by producing the peptide in the presence of uniformly labelled ^15^N‐^13^C‐proline. In MALDI‐TOF heavy proline‐labelled C5a^CF^ is detected at a molecular weight of 11600 Da corresponding to an 18 Da mass shift as expected for the incorporation of three ^15^N‐^13^C‐proline residues. We detect minor populations of unlabelled or partially labelled C5a^CF^, however, these always represented < 20% of the overall produced ligand as determined by comparing relative peak intensities of the unlabelled and labelled peptide. The presence of incompletely labelled C5a^CF^ species would allow a correction of the calculated concentration of [^3^H]‐C5a^CF^ and thus an enhancement of the determined binding parameters by up to 20%. However, due to the low amount of incompletely labelled peptide populations, the observed experimental variation and the standard errors of the binding parameters a correction of the dissociation constant of the peptide was not applied.

Alanine scanning studies of complement 5a have revealed that the disulphide‐bonded core region (residues 21–55) and the C‐terminal region of the peptide are indispensable for high‐affinity binding of C5a and full activation of the receptor 22, 30. [^3^H]‐C5a^CF^ displays comparable binding affinities as determined with commercially available ^125^I‐labelled C5a (Table 1), indicating correct folding and formation of cysteine bridges. As the N‐terminal region of C5a is less crucial for receptor interaction than the C‐terminus and the core region, the N‐terminal hexa‐histidine tag does not have to be removed for high‐affinity ligand binding. Due to its large size compared to other GPCR peptide ligands, the N‐terminus displays enough conformational flexibility so that the attached tags do neither sterically nor electrostatically hinder ligand binding.

In contrast, the smaller ET‐1 mainly interacts with its receptor via its hydrophobic residues (Tyr^13^, Phe^14^, Trp^21^) as determined by alanine‐scanning studies. The two disulphide bridges that connect Cys^1^–Cys^15^ and Cys^3^–Cys^11^ bring the N‐terminal part of the peptide into close vicinity of the main receptor interacting residues Tyr^13^, Phe^14^ and Trp^21^. 33 It is therefore necessary to remove any remaining purification tags before receptor‐binding studies. We chose furin as the suitable protease as it does not result in any remaining N‐terminal amino acids of the recognition site after proteolysis. Efficient cleavage of the peptide with furin could be demonstrated in MALDI spectrometry, detecting peaks at the expected size of the cleaved peptide after furin treatment. IAM labelling of ET‐1^CF^ did not yield any peaks at higher molecular weights, indicating the formation of two cysteine bridges (Fig. 5). ET‐1 isomers containing the non‐native disulphide pairs Cys^1^–Cys^11^ and Cys^3^–Cys^15^ bind with drastically reduced affinities to the endothelin receptors highlighting the importance of the correct formation of cysteine bridges 25. As [^3^H]‐pET‐1^CF^ displays binding parameters comparable to values determined with commercial [^125^I]‐pET‐1 and receptors expressed in native tissues, we can conclude that our cell‐free radioligand is produced as the native Cys^1^–Cys^15^, Cys^3^–Cys^11^ isomer and displays a fold comparable to endogenous ET‐1.

We have chosen two GPCR peptide ligands containing different numbers of cysteine bridges and displaying different folds to demonstrate the usability of our adapted cell‐free system. Peptide ligands activating GPCRs are often difficult targets to produce in *in vivo* as well as *in vitro* systems, as they are small, display folded domains or no structural constraints at all and often contain hydrophobic or amphiphatic patches that interact with the transmembrane core of the receptors. The production of fully functional tritium‐labelled GPCR ligands emphasizes the robustness of our cell‐free system and renders it suitable for the synthesis of peptides and proteins for different purposes. Apart from tritium‐labelled amino acids, the system allows the incorporation of other radiolabels such as ^35^S which have the advantage of higher specific activities, of heavy isotopes for NMR studies or fluorescently labelled amino acids. Applications of the produced peptides range from custom labelling of commercially unavailable radioligands to ligand screening and large‐scale production of labelled peptides for NMR experiments. The advantage of introducing radiolabels in cell‐free reactions is the small amount of radioactive material needed and the low contamination risk of reusable equipment. Our cell‐free system can further be adapted to other cell‐free expression modes such as the batch system to facilitate handling and reduce the consumption of starting materials. Taken together, our system is a versatile tool for the fast and economic production of custom‐labelled peptides and proteins for functional and structural studies.

## Materials and methods

### Ligands

NDT 9513727 (*N,N*‐Bis(1,3‐benzodioxol‐5‐ylmethyl)‐1‐butyl‐2,4‐diphenyl‐1*H*‐imidazole‐5‐methanamine, Tocris Bioscience, Bristol, UK); endothelin‐1 (CSCSSLMDKECVYFCHLDIIW, human, porcine, Tocris), H_2_N‐ChaW (Phe‐Lys‐Pro‐DCha‐Trp‐DArg, AnaSpec, Fremont, USA).

### Expression and purification of DsbA

The *Escherichia coli DsbA* cDNA was amplified from the pETM52 vector (European Molecular Biology Laboratory, Heidelberg, Germany) 49 by PCR. A StrepII tag was added to the *DsbA* 3′end and the resulting construct was cloned into pETM11 via 5′ XbaI and 3′ NotI restriction sites. The integrity of the resulting plasmid was verified by sequencing (Eurofins, Ebersberg, Germany). For protein production the plasmid was transformed into Rosetta‐2 cells (Merck Millipore, Darmstadt, Germany), overnight cultures were diluted 1 : 100 into fresh LB‐medium and grown at 37 °C, 150 rpm until an optical density (at 600 nm) of 0.4 was reached. Protein production was induced by the addition of 1 mm isopropyl β‐D‐thiogalactopyranoside (IPTG). Cultures were cooled to 30 °C and incubated for 4 h, 150 rpm. Cells were harvested by centrifugation (5000 ***g***, 20 min, 4 °C) and stored at −80 °C until further use. For purification, cell pellets from 2 L *E. coli* culture were thawed on ice, resuspended in 30 mL buffer A (20 mm Hepes‐NaOH, pH 7.6, 100 mm NaCl, 5% (w/v) glycerol supplemented with complete EDTA‐free protease inhibitors (Roche Applied Science, Mannheim, Germany), 1 mm PMSF (Carl Roth, Karlsruhe, Germany)) and lysed by ultrasonication (Branson Sonifier 250, Branson Ultrasonics, Danbury, USA). Nonlysed cells and debris were pelleted by centrifugation (41 000 ***g***, 30 min, 4 °C) and cleared lysates were incubated with 1‐mL bed volume Streptactin (Qiagen Streptactin SuperFlow Plus, Hilden, Germany) for 3.5 h at 4 °C under rotation. The resin was transferred into a 20‐mL gravity flow column and washed with 15 column volumes (CV) buffer A. The protein was eluted with 10 CV buffer B (buffer A supplemented with 2.5 mm d‐desthiobiotin (Sigma Aldrich, Steinheim, Germany)) and concentrated using 10 kDa molecular weight cut‐off (MWCO) concentrators (Amicon, Merck Millipore, Darmstadt, Germany). Glycerol was added to a final concentration of 10% (v/v) and protein concentration was adjusted to 5 mg·mL^−1^. DsbA was aliquoted, flash frozen and stored at −80 °C until further use. Protein purity was analysed by SDS/PAGE using the NuPAGE system according to manufacturer's instructions (Life Technologies, Darmstadt, Germany).

### Cell‐free synthesis of GPCR ligands

For the cell‐free production of endothelin‐1 and complement 5a the cDNAs encoding the respective ligands were cloned into a modified pIVEX cell‐free expression vector (received from G. Stier, Biochemistry Center, Heidelberg University) via 5′ NcoI, 3′ NotI restriction sites. Complement 5a is flanked by an N‐terminal 6x His tag followed by a tobacco etch virus (TEV) cleavage site. Endothelin‐1 contains an N‐terminal 6x His tag followed by a TEV and furin cleavage site. Expression of both constructs is controlled by a T7 promoter. Cell‐free synthesis was accomplished using a homemade S30 extract from *E. coli* 6. The extract was prepared from the *E. coli* A19 strain, described in detail in 6. The T7 RNA polymerase was produced and purified as published 50. Cell‐free reactions were executed in continuous exchange mode by using different sizes of D‐Tube^™^ dialyzers (Merck Millipore) (mini for reactions containing labelled amino acids and maxi for reactions with unlabeled amino acids) and a 1 : 17 ratio between reaction (RM) and feeding mix (FM). Mini D‐tube^™^ dialyzers were placed into 2 mL tubes filled with feeding mix, maxi dialyzers into 50 mL tubes, respectively. The optimized final reactions contained: 0.06% NaN_3_, 2.2% PEG8000, 120 mm potassium acetate, 14 mm magnesium acetate, 100 mm Hepes‐KOH pH 8.0, 1 tablet complete EDTA free protease inhibitors (Roche Applied Science) per 15.2 mL, 0.2 mm folinic acid, 2 mm reduced glutathione (GSH), 26 mm phosphoenolpyruvate, 26 mm acetyl phosphate, 5.4 mm (FM)/10.2 mm (RM) ATP, 3.6 mm (FM)/6.8 mm (RM) GTP/UTP/CTP, 1 mm (RM)/2 mm (FM) amino acids RCDWME, 0.5 mm (RM)/1.2 mm (FM) each of the remaining amino acids. The reaction mix was further supplemented with: 0.04 mg·mL^−1^ pyruvate kinase (Roche Applied Science), 0.52 mg·mL^−1^ *E. coli* tRNA (Roche Applied Science), 0.4 U μL^−1^ RiboLock RNase inhibitor (Thermo Scientific, Schwerte, Germany), 0.1 mg·mL^−1^ T7 RNA polymerase, 7 ng·μL^−1^ plasmid DNA, 75**–**100 μg·mL^−1^ purified DsbA and 40% (v/v) S30 extract. Reactions were incubated at 30 °C, 100 rpm in an Infors Multitron shaker incubator (Infors, Bottmingen, Switzerland) overnight. Cell‐free reactions were analysed by SDS/PAGE using 10% Bis/Tris Novex NuPAGE gels (Life Technologies).

### Purification of cell‐free synthesized proteins

Reaction and feeding mixes were pooled and applied to a 1 mL HisTrap crude FF column (GE Healthcare, Freiburg, Germany) using an Äkta system (GE Healthcare). Cell‐free synthesized C5a (C5a^CF^) was purified in buffer C^C5a^ containing 20 mm Hepes‐NaOH, pH 8.0, 100 mm NaCl, 5% glycerol supplemented with 1 mM PMSF. The column was washed with 5 CV buffer C1^C5a^ (buffer C^C5a^ supplemented with 20 mm imidazole), 5 CV buffer C2^C5a^ (buffer C^C5a^ supplemented with 50 mm imidazole) and eluted in 5 CV buffer D^C5a^ (buffer C^C5a^ supplemented with 400 mm imidazole). Cell‐free‐translated ET‐1 (fET‐1^CF^) was purified according to the same protocol, but buffer C^ET−1^ was composed of 20 mm Hepes‐NaOH, pH 7.0, 100 mm NaCl. Proteins were concentrated in 3 kDa MWCO amicons (Merck), buffer was exchanged to buffers C^C5a^ or C^ET−1^ by repeated concentration and dilution, proteins were flash frozen in liquid nitrogen and stored at **−**80 °C until use.

### Cell‐free synthesis of radioligands

For the cell‐free synthesis of radioligands one amino acid was replaced with a tritiated variant depending on its abundance in the respective ligand. Complement 5a was produced in the presence of 1 μm (FM)/2.1 μm (RM) L‐[2,3‐^3^H]‐proline (specific activity 55 Ci·mmol^−1^, Perkin Elmer, Boston, USA). For labelling of endothelin‐1 1 μm (FM)/0.9 μm (RM) L‐[2,3,4,5,6‐^3^H]‐phenylalanine (specific activity 128.1 Ci·mmol^−1^, Perkin Elmer) was added. The cell‐free reaction was initiated omitting the labelled amino acid and incubated at 30 °C, 100 rpm for 3 h. The reaction mix was removed from the D‐tube^™^ dialyzer and incubated with 0.2 mL Ni‐NTA magnetic agarose beads suspension (Qiagen) per mL reaction mix for 10 min to remove undesired side products translated with endogenously present amino acids from the S30 extract. The reaction mix was removed from the magnetic beads, supplemented with 0.25 mm L‐histidine to account for potential losses in the IMAC (immobilized metal ion affinity chromatography) step and the tritium‐labelled amino acid. The feeding mix was discarded and replaced with a fresh mix containing the labelled amino acid. Reactions were incubated at 30 °C in a water bath under constant shaking overnight.

### Purification of tritium‐labelled ligands

For purification of tritium‐labelled complement 5a ([^3^H]‐C5a^CF^), reaction and feeding mixes were pooled and applied to a 1 mL HisTrap FF column (GE Healthcare) equilibrated in buffer C^C5a^ using a peristaltic pump. The column was washed with 4 CV buffer C1^C5a^, 4 CV buffer C2^C5a^ and eluted in 4 CV buffer D^C5a^. Tritium‐labelled Endothelin‐1 ([^3^H]‐fET‐1^CF^) was purified accordingly with the following modifications: the peptide was purified in buffer C^ET−1^ using 0.5 mL bed volume Ni Sepharose HisTrap HP (GE Healthcare) in a gravity flow column. Buffer exchange to buffers C^C5a^ or C^ET−1^ was performed by repeated concentration and dilution in a 3 kDa MWCO concentrator (Amicon) and protein concentration was calculated using the specific activity of incorporated [^3^H]‐Pro or [^3^H]‐Phe residues. [^3^H]‐C5a^CF^ contains three proline residues, furin‐cleaved [^3^H]‐pET‐1^CF^ contains one phenylalanine residue.

### Furin‐cleavage of fET‐1^CF^

The full‐length fET‐1^CF^ peptide was cleaved with 20 U·mL^−1^ furin (New England Biolabs, Ipswich, USA) overnight at room temperature yielding processed ET‐1^CF^ (pET‐1^CF^). The reaction was terminated by the addition of 100 nm furin inhibitor I (Merck; 1 h incubation at RT) and subsequent flash freezing in liquid nitrogen. Peptides were stored at −20 °C until further use in binding assays.

### Iodoacetamide‐labelling assay

About 5–10 μg C5a^CF^ or ET‐1^CF^ (both in their respective buffers C^C5a^ or C^ET−1^ from purification) were labelled with 0.1 mm, 1 mm and 10 mm iodoacetamide (Thermo Scientific) for 15 min at RT.

### MALDI‐TOF

For molecular mass determination, protein preparations were mixed with DHB matrix solution (2,5‐dihydroxybenzoic acid, 300 mg·mL^−1^ DHB in a 1 : 2 mixture of water:acetonitrile containing 0.1% trifluoroacetic acid, Bruker Daltonics, Bremen, Germany) in a 1 : 1 (v/v) ratio and spotted on a ground steel target plate (Bruker Daltonics). Then MALDI‐TOF mass spectra were recorded in a mass range of 5–20 kDa using a Bruker Autoflex III Smartbeam mass spectrometer in linear mode. Detection was optimized for m/z values around 10 kDa and the spectra were calibrated using the near‐neighbour method (protein molecular weight calibration standard 1, Bruker Daltonics).

### GPCR production in *Sf*9 insect cells and membrane preparation

The complement 5a receptor was coproduced with Gαi2 in *Sf*9 insect cells. The cDNA encoding the human complement 5a receptor flanked by an N‐terminal 10x His and FLAG tag and a C‐terminal StrepII tag was cloned into the pOET5 transfer vector (Oxford Expression Technologies, Oxford, UK). pOET5 contains two expression cassettes, the receptor is expressed under the control of a polyhedron promoter; Gαi2 expression is regulated by a p10 promoter. Recombinant baculoviruses were generated with the flashBAC protein expression system (Oxford Expression Technologies) according to manufacturer's instructions. The human endothelin B receptor cDNA was cloned into the pVL transfer vector and flanked with N‐terminal 10x His and FLAG tags and a C‐terminal biotinylation domain. The receptor lacks the N‐terminal signal peptide (first 26 amino acids), the glycosylation site at N59 and the putative metallo‐protease site at R64 were mutated to alanine. Recombinant baculoviruses were generated using the BaculoGold expression system (Cell Concepts, Umkirch, Germany). *Sf*9 cells were cultured in TMN‐FH medium (c.c.pro, Oberdorla, Germany) supplemented with 2 mm glutamine (PAA Laboratories, GE Healthcare, Munich, Germany), 5% (v/v) foetal calf serum (BioWest, Nuaillé, France), 7.5 nm vitamin B12 (Sigma Aldrich), 50 μg·mL^−1^ gentamicin (Thermo Fisher Scientific). For suspension cultures 0.1% (v/v) Pluronic F68 (AppliChem, Darmstadt, Germany) was added to the culture medium. *Sf*9 cells were infected with baculoviruses encoding the respective receptors at a cell density of 2 × 10^6^ cells·mL^−1^ with a multiplicity of infection of 10 and incubated for 96 h at 27 °C. Cells were harvested by centrifugation and stored at −80 °C until further use. After resuspension in lysis buffer (50 mm Hepes‐NaOH, pH 7.6, 100 mm NaCl, 10 mm EDTA and protease inhibitors (complete EDTA free, Roche Applied Science); 1 mm PMSF (Carl Roth)) cells were lysed by nitrogen decompression (Parr Instrument Company, Moline, IL, USA). Nonlysed cells and debris were removed by centrifugation (1000 ***g***, 10 min, 4 °C) and membranes were subsequently pelleted by ultracentrifugation (180 000 ***g***, 90 min, 4 °C). Membranes were homogenized in membrane buffer (lysis buffer supplemented with 5% (w/v) glycerol), aliquoted, flash frozen and stored at −80 °C until use. Total membrane protein content was determined by the bicinchoninic acid (BCA) method using bovine serum albumin as the standard.

### Ligand‐binding assays

For the determination of binding parameters, membranes containing the respective receptors (10–50 μg total membrane protein) were incubated with cell‐free synthesized tritium‐labelled ligands in varying concentrations. Binding was terminated by rapid filtration over glass fibre filters pretreated with 0.3% polyethyleneimine using a Brandel harvester (Brandel, Alpha Biotech Ltd, Killearn, UK) and analysed by liquid scintillation counting. The endothelin B receptor binding assays were performed in 25 mm Hepes‐NaOH, pH 7.4, 10 mm MgCl_2_, 1 mm CaCl_2_, 0.5% BSA, incubated 60 min at RT, filtrated over GF/C glass fibre filters and washed with 50 mm Hepes‐NaOH, pH 7.6. C5aR binding assays were incubated for 120 min in 50 mm Hepes‐NaOH, pH 7.4, 5 mm MgCl_2_, 1 mm CaCl_2_, 0.5% BSA, 75 mm NaCl at room temperature, terminated by filtration over GF/C filters and washed with 50 mm Hepes‐NaOH, pH 7.6, 500 mm NaCl. For the determination of dissociation constants of the cell‐free synthesized radioligands, membranes were incubated with increasing concentrations of radioligands (1 pM‐1 nm for [^3^H]‐pET‐1^CF^ and 31 pM‐8 nm for [^3^H]‐C5a^CF^). Nonspecific binding was assessed in the presence of 1 μm unlabelled endothelin‐1 or 50 μm unlabelled H_2_N‐ChaW in case of ET_B_R or C5aR respectively. Inhibition constants of unlabelled ET_B_R or C5aR ligands were determined by incubating membranes with a constant concentration of cell‐free synthesized radioligand (0.1 nm for [^3^H]‐pET‐1^CF^ and 2 nm for [^3^H]‐C5a^CF^) and increasing concentrations of unlabelled ligands (3 pM‐31 nm for endothelin‐1, 0.03 nm‐10 μm for NDT 9513727 and 0.3 nm‐100 μm for H_2_N‐ChaW).

### Statistical analysis

Data are represented as means ± SEM from three or two independent experiments performed in triplicate and analysed in graphpad prism6 (GraphPad Software, La Jolla, CA, USA). IC_50_ values were converted into inhibition constants (K_I_) via the Cheng–Prusoff 51 equation using the determined dissociation constants (K_D_) and the applied ligand concentrations ([L]):

$$K_{I}=\frac{{\text{IC}}_{50}}{1+\frac{\left[L\right]}{K_{D}}}$$

## Author contributions

LJ, RT, JDL performed experiments, LJ, RT, JDL, JP planned experiments, LJ, JDL, JP analysed data; LJ, JDL, JP wrote the paper; HM supervised the overall project.

## Supporting information


**Data S1.** Methods.
**Fig. S1.** SDS/PAGE of purified *Escherichia coli* DsbA.
**Fig. S2.** Endogenous amino acids in the S30 extract lead to the translation of C5a^CF^ in the absence of proline.
**Fig. S3.** Iodoacetamide labelling of lysozyme does not result in significant shifts of the molecular mass.
**Fig. S4.** Uniformly ^15^N^13^C‐labelled proline is efficiently incorporated into C5a^CF^.
