# Supplementary material for: Cell‐free synthesis of isotopically labelled peptide ligands for the functional characterization of G protein‐coupled receptors
Source: FEBS Open Bio. 2015 Dec 29;6(1):90–102. doi: 10.1002/2211-5463.12008 (PMC4794788; doi:10.1002/2211-5463.12008)
Supplement: Supplementary file 1 — Data S1. Methods. Fig. S1. SDS/PAGE of purified Escherichia coli DsbA. Fig. S2. Endogenous amino acids in the S30 extract lead to the translation of C5aCF in the absence of proline. Fig. S3. Iodoacetamide labelling of lysozyme does not result in significant shifts of the molecular mass. Fig. S4. Uniformly 15N13C‐labelled proline is efficiently incorporated into C5aCF. [file FEB4-6-90-s001.docx]

**Supporting information**

**Supplementary methods**

**Western Blot**

Gels were blotted using the iBlot system (Life Technologies, Darmstadt, Germany) and probed with an anti-penta histidine antibody (Qiagen, Hilden, Germany) coupled to alkaline phosphatase.


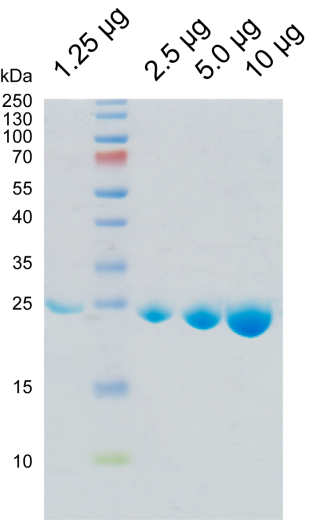


**Figure S1 – SDS PAGE of purified *E. coli* DsbA.** Increasing amounts of DsbA (1.25 – 10 µg) were analyzed on a 12% BisTris NuPAGE gel (Life Technologies) to verify the purity of DsbA. The protein is > 90% pure, as no contaminations are visible even when overloading the gel with 10 µg DsbA.


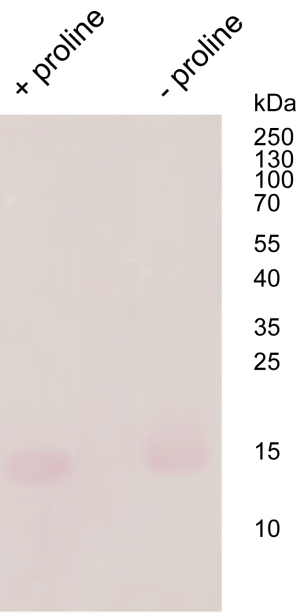


**Figure S2 – Endogenous amino acids in the S30 extract lead to the translation of C5a^CF^ in the absence of proline.** The cell-free translation of C5a^CF^ was executed in the presence of all amino acids (left lane, + proline) and in the presence of all amino acids except proline (right lane, - proline). The reactions were subsequently analyzed on a Western blot using an anti-penta histidine antibody. Both cell-free reactions result in the translation of C5a^CF^.


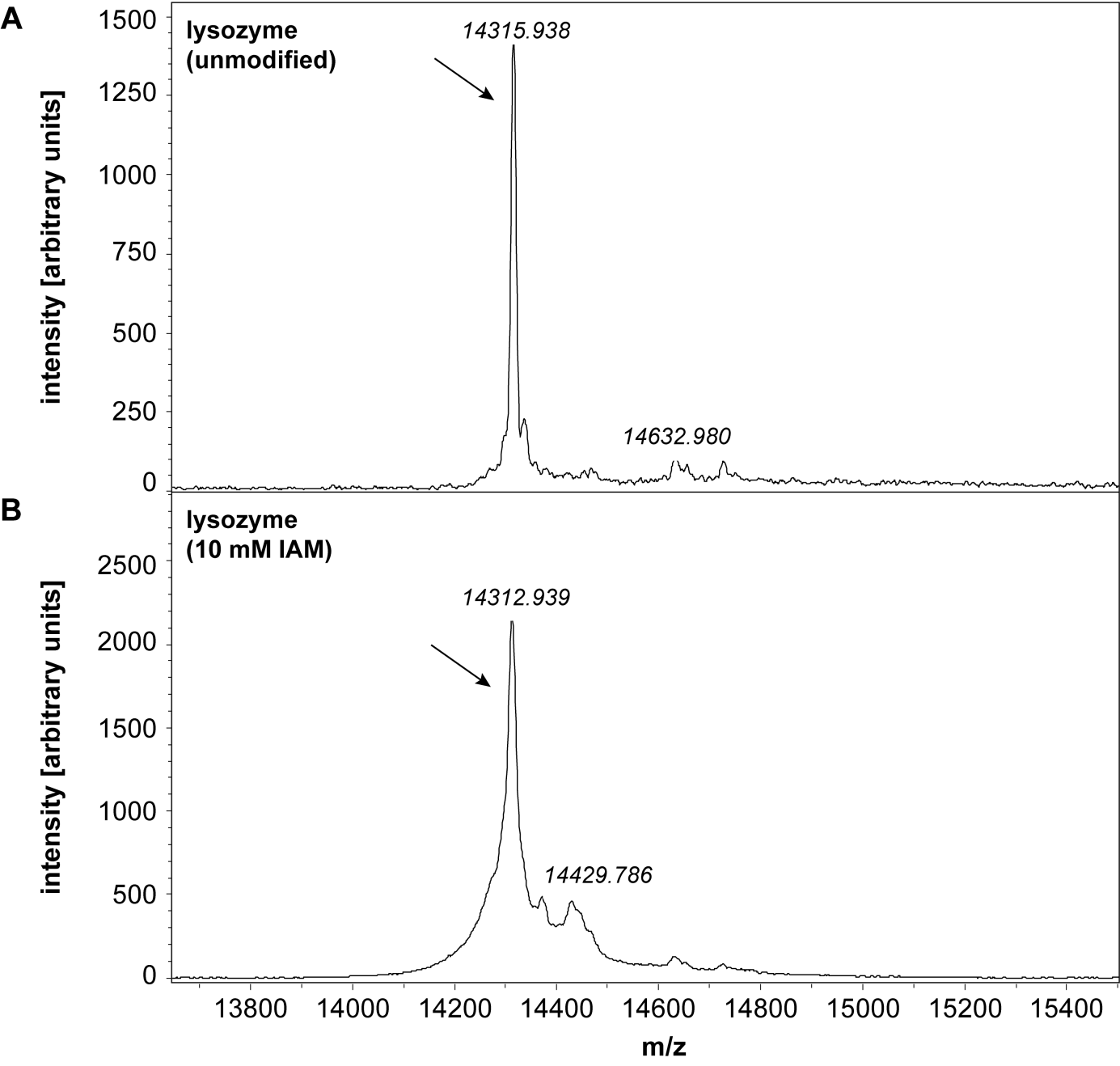


**Figure S3 – Iodoacetamide labelling of lysozyme does not result in significant shifts of the molecular mass.** Native lysozyme contains four disulfide bonds and no reduced cysteine residues. A, Unmodified lysozyme (Hampton Research, Aliso Viejo, USA) is detected at 14315 Da corresponding to the theoretical molecular weight of 14313 Da. B, Upon treatment with 10 mM iodoacetamide (IAM) only minor peaks can be detected at higher molecular weights, confirming that IAM modifications do not occur non-specifically.

**
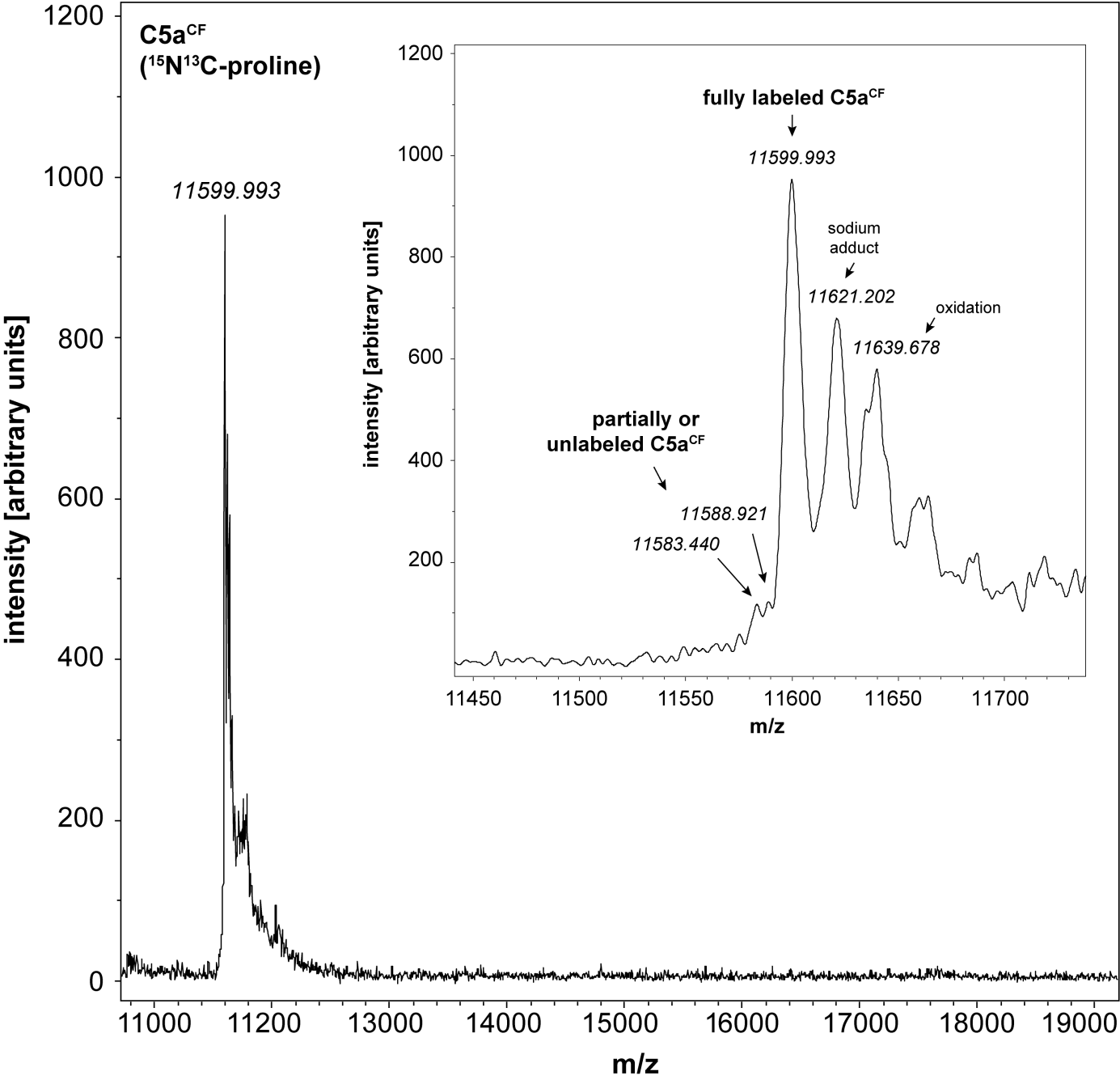
**

**Figure S4 – Uniformly ^15^N^13^C labeled proline is efficiently incorporated into C5a^CF^.** C5a^CF^ has a theoretical molecular weight of 11582 Da which is expected to increase by 18 Da upon incorporation of three ^15^N^13^C-labeled proline residues. After production of C5a^CF^ in the presence of ^15^N^13^C-proline, the peptide is detected at 11600 Da in MALDI-TOF, corresponding to the expected 18 Da mass shift of a fully labeled peptide. Only minor amounts (less than 20%) of unlabelled or partially labeled C5a^CF^ variants can be detected. The peaks at higher molecular weights presumably represent sodium adducts and oxidized species of C5a^CF^.
